# Age-related differences in hippocampal network engagement during safety processing in adolescents

**DOI:** 10.1016/j.dcn.2026.101791

**Published:** 2026-07-28

**Authors:** Yubing Zhang, Madeline K. Coates, Marta I. Garrido, Sarah M. Tashjian

**Affiliations:** a School of Psychological Sciences, The University of Melbourne, Melbourne, VIC 3010, Australia; b Graeme Clark Institute for Biomedical Engineering, The University of Melbourne, Melbourne, VIC 3010, Australia

**Keywords:** Adolescence, FMRI, Hippocampus, Safety, Threat

## Abstract

Adolescence is a period characterized by exploration, altered risk-taking, and increased vulnerability to mental health disorders. These phenomena may reflect underlying challenges in safety evaluation. Successfully navigating adolescence may therefore be related to the maturation of neural circuits that support safety evaluation, yet how these mechanisms function during development remains unclear. Using 7-Tesla functional magnetic resonance imaging, we recorded neural response in 33 adolescents (*M*_Age_ = 14.88 years, 19 females) during evaluation of threat (external cues that signal potential danger) and protection (resources available to an agent that increase safety). Our findings reveal age-related differences in neural recruitment during accurate estimation of protection, such that younger adolescents (12–14 years) exhibited greater hippocampal engagement, whereas older adolescents (15–17 years) exhibited a more integrated circuit involving the hippocampus and anterior ventromedial prefrontal cortex (vmPFC). Our results also provide insight into how competition between threat and protection is resolved within the visual cortex during adolescent safety evaluation, demonstrating enhanced perceptual sensitivity to protection signals compared to threat. Behavioral analysis across a broader developmental spectrum (*N* = 63, *M*_*Age*_ = 24.18 years, range 12–40 years, 34 females, including adults from prior work) revealed a quadratic association between age and protection estimation accuracy, with lower accuracy in mid-to-late adolescence relative to early adolescence and adulthood. Together, our behavioral and neural results indicate adolescence is an important developmental period for safety processing, particularly with respect to accurately estimating safety.

## Highlights

• Safety estimates are more influenced by protection than threat.
• Hippocampal activation relates to protection accuracy in younger adolescents.
• Hippocampal-vmPFC connectivity relates to protection accuracy in older adolescents.
• Greater perceived protection is associated with stronger visual processing.

## 1 Introduction

Safety evaluation can be defined as the comprehensive process of assessing one’s safety through the detection, prediction, and integration of safety information to guide behavior (Tashjian et al., 2025). The ability to accurately evaluate safety is important for supporting adaptive behaviors, such as exploration and learning. Accurately evaluating safety may be especially challenging during adolescence for multiple reasons (Bach and Dayan, 2017, LeDoux and Pine, 2016). First, the adolescent period requires navigating novel environments to gain independence and experience, which may result in increased uncertainty with respect to safety (Crone and Dahl, 2012, Gopnik et al., 2017, Murty et al., 2016, Somerville et al., 2017, Spear, 2000). Second, adolescents undergo crucial neurobiological changes (e.g., prefrontal-limbic development), which can impair safety evaluation due to its reliance on bottom-up as well as top-down neural processes (Larsen and Luna, 2018, Hartley and Somerville, 2015, Towner et al., 2023). These features of adolescent development may help explain the onset of mental health disorders and risky behaviors during this period. Adolescence is a peak period for the emergence of anxiety disorders and related mental health difficulties, which often involve biased judgments about safety (Casey et al., 2008, Ciranka and van den Bos, 2021, Lee et al., 2014). Adolescence is also marked by altered risk-taking behaviors, including increased reckless driving and substance abuse, which similarly involve challenges in evaluating safety and the potential consequences of unsafe choices (Hartley and Somerville, 2015, Galván and Rahdar, 2013).

**Fig. 1:**
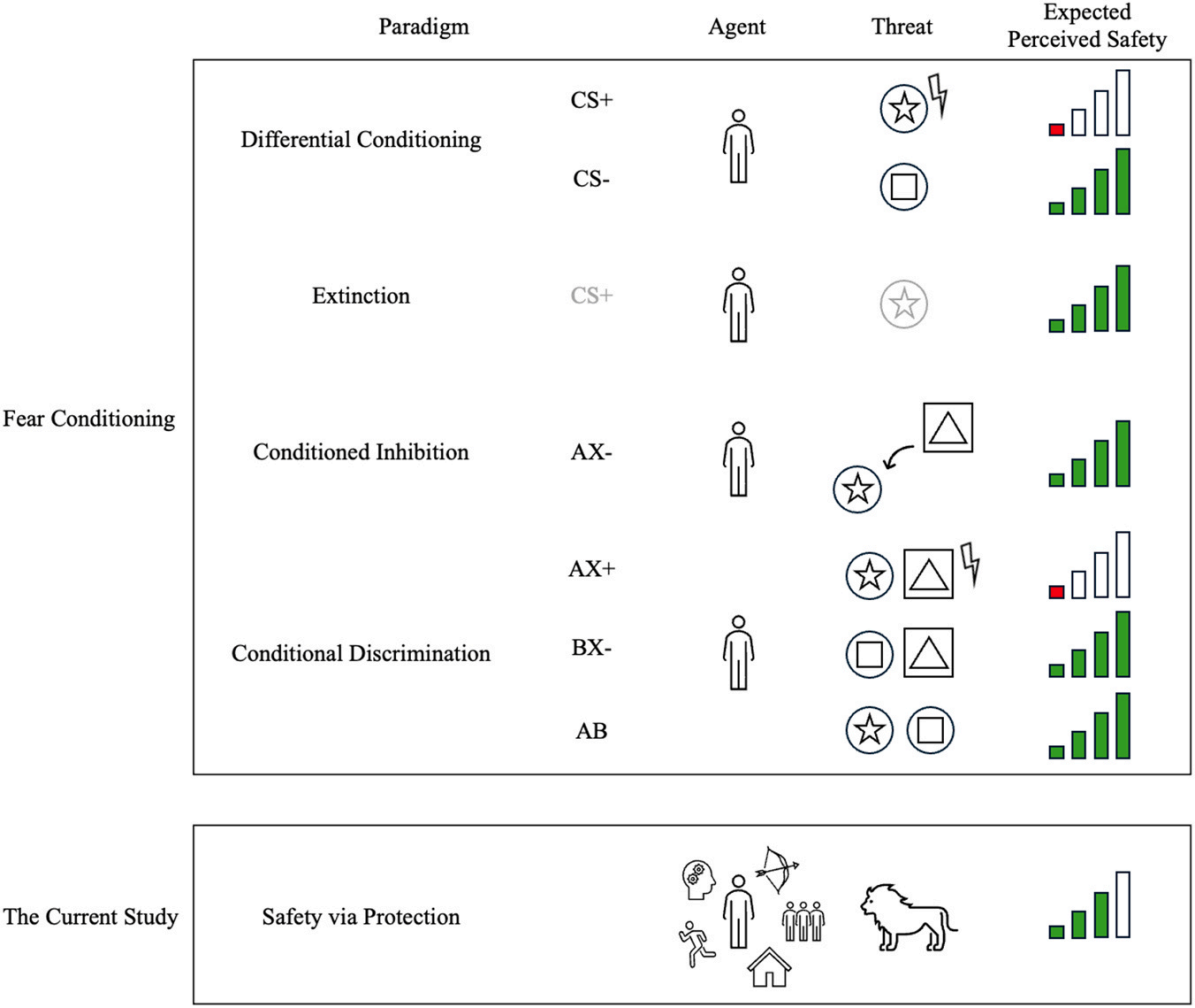
Comparison of traditional fear conditioning paradigms with the current study. Expected perceived safety reflects the safety perception participants would have if they accurately learned the task contingencies. In fear conditioning, safety is increased by modifying the threat through an unrelated neutral cue (differential conditioning, CS-), threat neutralization (extinction), or the addition of a modifier that signals threat neutralization (conditioned inhibition and conditional discrimination). In the current study, safety is increased by modifying resources at the agent’s disposal (through protection) without modifying the threat.

To date, most of what is known about safety evaluation stems from fear conditioning research (Grasser and Jovanovic, 2021, Sangha et al., 2020), although definitions of safety subtly differ across paradigms (see Fig. 1). Fear conditioning paradigms are used to understand both threat and safety learning. In differential fear conditioning, individuals learn that the absence of an aversive outcome signals safety. In this paradigm, a neutral conditioned stimulus (CS+) is paired with an aversive unconditioned stimulus (US, e.g., shock), while another conditioned stimulus (CS-) is not paired with the US. Over time, the CS + elicits conditioned responses (CR, e.g., freezing), whereas the CS- serves as a safety signal. During extinction, the CS + is no longer paired with the US, such that the previously threatening stimulus is neutralized and comes to signal safety. In conditioned inhibition, while stimulus A alone indicates threat, when combined with a second, neutral stimulus X (AX-), the expected threat is neutralized and the AX- pair serves to signal safety. In conditional discrimination, AX is paired with the US (AX+) and BX is paired with the absence of the US (BX-), such that presenting A with B reduces fear responses relative to AX + , reflecting transfer of safety from B to A.

Although these fear conditioning paradigms are well established, they may introduce interpretive challenges when applied to adolescents. First, evidence from fear conditioning is mixed regarding how safety evaluation develops during adolescence. Some studies suggest age-related differences between childhood and adolescence, with behavioral and neural responses appearing more adult-like by adolescence (Abend et al., 2020, Britton et al., 2013, Harrewijn et al., 2021, Waters et al., 2017, Widegren et al., 2025). By contrast, other studies suggest an adolescent-specific reduction in safety evaluation (Ganella et al., 2018, Lau et al., 2011, Pattwell et al., 2012). These discrepancies may stem from potential inconsistencies in how ‘safety’ is conceptualized. In differential conditioning and extinction, safety is represented as a CS- (or extinguished CS+) that signals the complete absence of threat, whereas in conditioned inhibition and discrimination, safety is represented as the active suppression of threat through adding a safety stimulus (Laing and Harrison, 2021, Laing et al., 2022). Second, fear conditioning paradigms typically use arbitrary neutral stimuli (e.g., shape, tone, face with neutral expression) that require associative learning. Associative learning is clinically important for understanding how neutral cues become imbued with fear and drive maladaptive avoidance. However, these paradigms may depend on cognitive processes that continue to mature during adolescence (e.g., working memory; Luciana et al., 2005). Thus, developmental differences in safety evaluation may be confounded with the development of general cognitive abilities. Moreover, fear conditioning paradigms typically define safety as the absence or modification of external threat cues, which may be less representative of adaptive threat responding in real-world contexts involving physical danger. For example, fear of lions should not be extinguished entirely in the real world. Little work has examined safety processing outside of conditioning paradigms (Fullana et al., 2020, Odriozola and Gee, 2021), leaving gaps in understanding the development of and mechanisms underlying safety processing.

To address these gaps, the current study adopts Tashjian and colleagues’ (2025) multi-dimensional definition of safety, which frames safety evaluation as emerging from the integration of information about threat and protection (Fig. 1). Protection refers to resources available to an agent that increase safety. This distinction becomes clear when considering real-world scenarios. For example, imagine confronting a venomous snake with bare hands versus with a hiking pole to maintain distance. In both scenarios, the threat is unchanged; however, perceived safety increases when protection (the hiking pole) is present. This framework considers safety evaluation as distinct from conditioning conceptualizations, although it is most comparable to conditioned inhibition. In conditioned inhibition, the safety cue is typically an arbitrary inhibitor with no inherent value (e.g., Laing et al., 2021), whereas protection has its own intrinsic safety value. Both inhibitory cues and protection can reduce the expected danger associated with a threat without necessarily altering its learned threat value (in contrast to extinction), but only protection does so in a probabilistic, resource-based manner (Harrewijn et al., 2021, Tashjian et al., 2025). The current protection framework captures real-world contexts involving actual physical danger, where safety is often estimated in the continued presence of threat and complete fear reduction may not be adaptive (Tashjian et al., 2021). This differs from traditional fear conditioning paradigms, which are often used to understand mechanisms relevant to the reduction of disproportionate fears, including social fears such as fear of public speaking, that can interfere with daily functioning despite posing little direct physical danger (Anderson et al., 2013, Beidel et al., 2014). Moreover, all cues in the current study possess inherent, universally recognized values, allowing participants to draw on pre-existing knowledge rather than learning artificial associations. Using recognizable cues is particularly valuable in adolescent research, as it reduces learning confounds and ensures that age-related differences more directly reflect safety processing rather than other executive functions.

Due to ongoing cognitive development, adolescents may face unique challenges in evaluating safety. Compared to threat detection, safety evaluation is cognitively more complex because it requires integrating threat-related sensory inputs with self-relevant considerations inherent to protection. This integrative judgment may be supported by adolescents’ increasing capacity for nuanced judgments and meta-cognition (Edelson and Reyna, 2021, Kveraga et al., 2007, Weil et al., 2013). Simultaneously, the ability to identify and use protective factors likely requires cognitive functions such as working memory and abstract thinking, which continue to mature during adolescence (Dumontheil, 2014, Luciana et al., 2005). Consequently, difficulties in integrating protection estimations with threat estimations may contribute to challenges in safety evaluation that have yet to be identified.

Neurodevelopmental changes occur alongside adolescent cognitive changes, as indicated by different developmental trajectories for neural systems supporting safety processing. Animal and human studies suggest that threat and protection are processed in distinct neural networks, which together facilitate safety evaluation (Kong et al., 2014, Tashjian et al., 2025, Wen et al., 2024). Threat processing involves the defensive circuit, including the periaqueductal gray, amygdala, insula, hypothalamus, and the posterior ventromedial prefrontal cortex (vmPFC; Greco and Liberzon, 2016; Mobbs et al., 2020; Tovote et al., 2016). In contrast, protection processing engages the hippocampus, anterior cingulate cortex (ACC), and anterior vmPFC. The hippocampus encodes successful confrontations of threat during extinction and supports strategic defensive decisions (Micale et al., 2017, Qi et al., 2018). The hippocampus also compares new inputs with stored memory patterns of danger during generalization, promoting pattern separation when stimuli are dissimilar to enhance safety estimations (Lissek et al., 2014). The ACC inhibits fear responses and allows context-dependent adjustment of safety responses (Jovanovic et al., 2013, Wu et al., 2023). The anterior vmPFC contributes to extinction retention, protection evaluation, and stress inhibition, all of which are key for safety evaluation (Battaglia et al., 2022, Harrison et al., 2017, Maier et al., 2006, Phelps et al., 2004, Schiller et al., 2008, Tashjian et al., 2021). The significant change in these neural networks during adolescence may create unique challenges for safety evaluation. In particular, the hippocampus and ACC mature earlier than the vmPFC (Casey et al., 2016, Fuster, 2002, Lau et al., 2011, Mills et al., 2014), creating imbalances that may influence adolescent safety evaluation. Examining safety processing through an integrated threat and protection framework is essential to understand how developing neural circuits differentially support the multi-dimensional nature of safety evaluation.

The current study aims to expand and clarify the understanding of safety processing during adolescence. Our paradigm incorporates threat and protection cues that are integrated to convey an overall safety estimate. We employed 7-Tesla functional magnetic resonance imaging (7 T fMRI) to achieve high resolution for identifying the neural systems involved. Compared to the current standard 3 T fMRI, 7 T fMRI can provide improved temporal signal-to-noise ratio and increased signal sensitivity (Torrisi et al., 2018, Viessmann and Polimeni, 2021). These improvements may facilitate detecting subtle age-related effects, especially in small subcortical and midline prefrontal regions known to change during development (Morris et al., 2019). Only a limited number of studies have investigated safety processing during adolescence (e.g., Gold et al., 2020; Abend et al., 2020). Of those, most test adolescents as a homogeneous group compared to adults. By contrast, our study examines age-related neural and behavioral differences within the adolescent sample. We hypothesized that: (a) protection recruits distinct neural systems from those engaged by threat; (b) safety evaluations vary with stimulus safety probability, sequence of stimulus presentation, and stimulus type (threat versus protection), with protection exerting greater influence; (c) because our task requires higher-order cognitive processes such as information integration, we expected age-related differences, with younger adolescents demonstrating less accurate safety estimation; (d) although the vmPFC has been identified as a central hub for safety evaluation in adults, it continues to mature throughout adolescence. Therefore, we expected adolescents to recruit subcortical systems to a greater extent during safety evaluation, particularly at younger ages.

## 2 Methods

### 2.1 Participants and Ethics

Thirty-five participants aged 12–17 years were recruited through flyers and online postings. Our inclusion criteria were as follows: (1) not claustrophobic; (2) no metal contraindications; (3) not pregnant; (4) no hearing or sight difficulties; (5) no medicated neurological or psychiatric conditions; and (6) fluent in verbal and written English. Two participants were excluded from all analyses, one due to an incomplete MRI session and one due to a potential neurological disorder identified during scanning. This resulted in a final sample of 33 participants (*M*_*Age*_ = 14.88 years, *SD*_*Age*_ = 1.47; 19 females, 58% of the sample). All methodology was approved by the University of Melbourne Human Research Ethics Committee (27613). Participation was voluntary, and participants were compensated for their time. Informed assent was obtained from adolescents, and informed consent from their parent/guardian.

To determine the appropriate sample size for the current study, we conducted a power analysis using data from a previous investigation in adults using the same experimental paradigm (Tashjian et al., 2025). In that study, significant activation in the anterior vmPFC for protection compared to threat was detected with a t-statistic of 4.08, corresponding to Cohen’s *d* = .74. Power analysis indicated that a sample size of *N* = 17 would achieve 80% power to detect activation differences of similar magnitude (*α* =.05, two-tailed). Due to the anticipated increase in heterogeneity with adolescents, we doubled the sample size for the current study.

For behavioral analyses, we combined our adolescent sample with the adult sample reported in Tashjian et al. (2025) to extend our investigation into early adulthood (adults: *N* = 30, *M*_*Age*_ = 27.83 years, *SD*_*Age*_ = 4.86, range 20–40 years, 15 females, 50% of the sample; full sample: *N* = 63, *M*_*Age*_ = 24.18 years, *SD*_*Age*_ = 7.23, range 12–40 years, 34 females, 54% of the sample). The present study used 7 T fMRI, which differs from the 3 T fMRI used by Tashjian et al. (2025) and may offer advantages for detecting subcortical activation. Consequently, all fMRI analyses reported herein were restricted to the adolescent sample (12–17 years) to avoid potential comparability issues.

### 2.2 Procedure

Prior to scanning, participants completed a series of online questionnaires. The MRI session consisted of a structural scan and three fMRI tasks, one of which was the Safety Evaluation Task described here. The other two tasks are out of scope and will be described elsewhere. For the Safety Evaluation Task, participants received task instructions and completed five practice trials during the structural scan. Participants then completed four functional runs of the task (approximately 6 min each).

### 2.3 Task Design

**Fig. 2:**
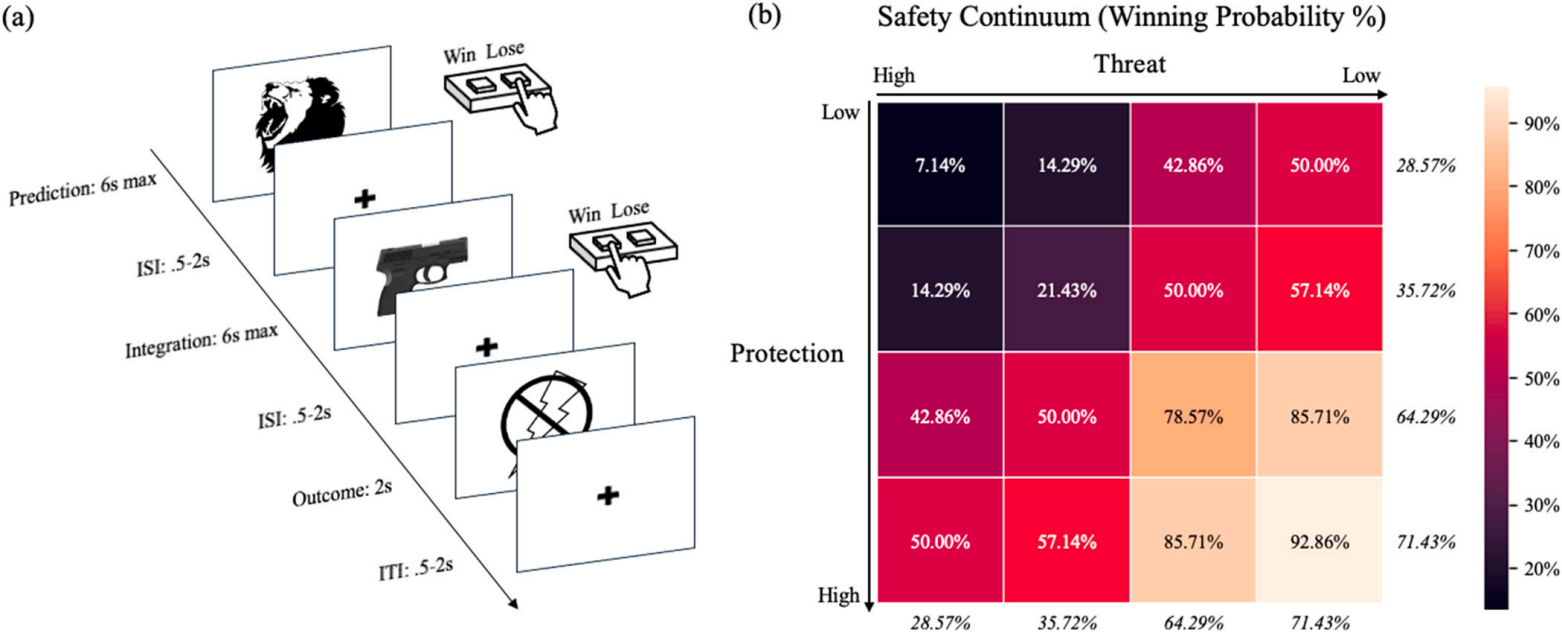
(a) An example trial of the Safety Evaluation Task. Participants saw an animal-weapon pair with each cue presented in isolation and in counterbalanced order. Participants were instructed to make binary choices about whether they thought they would win or lose the battle by pressing a button while the stimulus was presented. Choices were made in response to the first and second cue presentations. Participants saw the outcome of the battle for 2 s, which was paired with either a loud, unpleasant white noise (loss) or no noise (win). (b) The predetermined experimental safety continuum showing the probability of winning for each condition (protection continuum order: fist, stick, gun, grenade; threat continuum order: cat, goose, lion, grizzly bear). Values inside each grid cell indicate the probability for the animal-weapon pairing, while values in italics indicate the mean safety probability for each cue averaged across all pairings. The safety continuums were designed to be identically balanced. For example, participants had a 50.00% chance of winning with a combination of the most protective weapon and the most threatening animal, which was identical to the probability with a combination of the least powerful weapon and the least threatening animal. Similarly, the least protective weapon (fist, 28.57%) had an identical safety probability as the most threatening animal (grizzly bear, 28.57%), and the same for the most protective weapon (grenade, 71.43%) compared to the least threatening animal (cat, 71.43%).

The Safety Evaluation Task was adapted from prior work examining safety processing in adults (Tashjian et al., 2025). Participants were instructed to imagine they were fighting fictitious battles against threatening animals (cat, goose, lion, or grizzly bear) using protective weapons (fist, stick, gun, or grenade). Each cue (animal or weapon) was presented in isolation, and the order of presentation was counterbalanced. Thus, the first cue was presented without knowledge of the second cue. The second cue completed the information for the given trial. Participants were asked to make binary choices about whether they thought they would win or lose the battle against each animal using the weapon provided. Choices were made twice on each trial, in response to the first and second cue presentations (Fig. 2a). Each weapon/animal cue was shown for 6 s maximum (offset to participant button press), followed by a jittered 0.5- to 2-second interstimulus interval (ISI). After both cues were presented, participants saw the outcome (win/loss) for 2 s, which was paired with either a loud, unpleasant white noise (loss) or no noise (win). Trials were separated by an intertrial interval (ITI), presented for 0.5–2 s, with duration jittered. All participants completed 160 trials, with each animal-weapon pair presented 10 times. The task was programmed using PsychoPy version 2023.1.2.

Importantly, the outcomes of the trials were unrelated to participant choices and instead were experimentally predetermined. The safety probabilities were designed to be balanced (Fig. 2b), such that when presented in isolation (first cue presentation), the continuums of average safety probabilities were identical for weapons and animals. When presented together (second cue presentation), the pairs were also on identical continuums, such that participants had the same probability of winning whether equipped with the most powerful weapon while facing the most threatening animal or equipped with the least powerful weapon while facing the least threatening animal.

Stimulus development tests were conducted prior to data collection with a sample of 58 participants (*M*_*Age*_ = 23.07 years, *SD*_*Age*_ = 4.50, range = 18–38; 39 females, 67% of the sample) after excluding two who failed attention checks. Participants completed paired, head-to-head choices for 20 animals and 20 weapons. For each animal pairing, participants indicated which animal would win in a battle; for each weapon pairing, they indicated which weapon was more powerful. Additionally, participants rated the danger of each animal and the power of each weapon on a 0–100 scale (0 = not at all dangerous/powerful, 100 = extremely dangerous/powerful). Based on these results, four animals (cat, goose, lion, grizzly bear) and four weapons (fist, stick, gun, grenade) were selected to represent the high and low ends of the safety continuum. Full details of the stimulus development process are reported in Tashjian et al. (2025).

### 2.4 MRI Data Acquisition

Structural and functional MRI data were acquired at the Melbourne Brain Centre Imaging Unit (MBCIU) using a Siemens Magnetom 7 T Plus scanner equipped with a 32-channel head coil. Structural (T1-weighted) images were obtained using a magnetization-prepared 2 rapid acquisition gradient echo sequence (MP2RAGE; Marques et al., 2010): TR = 5000 ms, TE = 2.04 ms, flip angle 1 = 4°, flip angle 2 = 5°, field of view = 240 mm, slice thickness = 0.75 mm. Functional (T2-weighted) images were collected using a gradient echo-planar imaging sequence (EPI; Moeller et al., 2010): TR = 800 ms, TE = 22.20 ms, flip angle = 45°, field of view = 208 mm, slice thickness = 1.60 mm. A total of 84 interleaved slices were acquired parallel to the anterior-posterior commissure line with a multiband acceleration factor of 6. To correct for susceptibility-induced distortion, a short EPI sequence with reversed phase-encoding direction (posterior-anterior) was acquired with identical spatial parameters (5 volumes, same resolution).

### 2.5 Data Analysis

#### 2.5.1 Behavioral Analyses

Behavioral data analyses were performed using R version 4.5.1 (R Core Team, 2021). We separately examined participants’ win/lose choices in response to the first and second cue presentations. The first choice (safety prediction) reflects how participants estimate safety based on the type of cue (animal versus weapon). The second choice (safety integration) reflects how participants integrated information from both cues (animal and weapon combined).

We analyzed choice behavior (0 = lose, 1 = win) using mixed-effects logistic regressions with random intercepts and random slopes at the participant level. The general model structure was as follows: choice ∼ predictors + (1 + predictors | participant). Predictors included stimulus safety probability and stimulus type. Stimulus safety probability refers to the experimentally predetermined probability of winning given the information available at each cue. For the first cue presentation, the safety probability is the mean probability for that stimulus averaged across pairings (i.e., values in italics in Fig. 2b). For the second cue presentation, the safety probability is the probability for the weapon-animal pairing considering both cues presented during the trial (i.e., values inside each grid cell in Fig. 2b). Stimulus type refers to cue category (animal versus weapon, dummy-coded with weapon as the baseline).

We also analyzed change behavior (0 = no change, 1 = change) using the same method. Predictors include stimulus order and the change in safety probability. Stimulus order refers to the sequence of cue presentation (weapon or animal presented first followed by the other cue, dummy-coded with weapon first and animal second as the baseline). Change in safety probability was computed on each trial as the difference between the experimentally predetermined safety probability for the weapon-animal pairing and the mean safety probability associated with the first cue presentation. For example, if a grizzly bear is presented as the first cue, its safety probability is 28.57% (Fig. 2b). When a grenade is presented as the second cue, the grizzly bear-grenade pairing has a safety probability of 50.00%. Thus, the change in safety probability is 21.43%, indicating an increase in safety probability from presentation of the first cue (grizzly bear) to presentation of the second cue (grenade), given the second cue reflects the combination of both cues (grizzly bear + grenade)*.*

To examine how different animal-weapon pairings affect participants’ safety ratings, we classified all conditions into four categories: high protection and high threat, high protection and low threat, low protection and high threat, and low protection and low threat. Because the objective winning probability is 50% for both “high protection and low threat” and “low protection and high threat” scenarios, they are directly comparable. We therefore conducted a paired-samples *t*-test on participants’ subjective ratings for these two scenarios. We also calculated safety bias as the difference between participants’ subjective ratings and the objective winning probabilities across all four scenarios, and fitted a linear mixed-effects model with a random intercept at the participant level, including protection level, threat level, and their interaction as predictors of safety bias.

We further calculated participants’ safety estimation accuracy for each animal-weapon pairing as the difference between the proportion of trials they predicted as wins and the experimentally established probability of winning. Linear and quadratic regression analyses were conducted to test the effects of age on accuracy in different task conditions.

#### 2.5.2 MRI Analyses

MRI data were pre-processed using fMRIPrep version 23.2.1 (Esteban et al., 2019) and analyzed using FSL version 6.0.7.13 (Jenkinson et al., 2012). Structural scans were corrected for intensity non-uniformity, skull stripped, and normalized to the MNI ICBM 152 Nonlinear Asymmetrical template (2009c) through nonlinear registration. Functional scans underwent motion correction (24 parameters including 6 standard and 18 extended derivatives), susceptibility distortion correction, and co-registration to the T1-weighted reference image using boundary-based registration.

A first-level general linear model (GLM) was defined for each run of the Safety Evaluation Task with 11 regressors modeling different trial components. Regressors 1–7 modeled cue presentations: (1) Threat First (animal presented as the first image), (2) Protection First (weapon presented as the first image), (3) Threat Second, (4) Protection Second, (5) Win Outcome, (6) Lose Outcome, (7) Fixation (ITI + ISI). Regressors 8–11 modeled parametric modulation of cue presentations (regressors 1–4), using the experimentally predetermined win/lose probability as parametric modulators (Fig. 2b). Both standard and parametric regressors were modeled with a canonical double-gamma hemodynamic response function (HRF) for a duration from image onset to offset. Parametric modulation regressors were mean-centered and orthogonalized with respect to the lower-order regressors (Mumford et al., 2015). By including parametric modulation, we were able to identify brain regions where activation correlates with the safety probability rather than just the presence or absence of the stimulus. Temporal derivatives were included for all regressors to reduce slice-timing differences and variability in the HRF delays across regions, thereby enhancing model fit. The four runs of the Safety Evaluation Task were combined for each participant using a fixed-effect voxel-wise second-level model in FEAT.

Group-level analyses were performed using the FMRIB Local Analysis of Mixed-Effects (FLAME1) module in FSL (Beckmann et al., 2003). *Z*-statistic images were thresholded using a cluster-forming threshold of *Z* > 3.1 and a familywise error-corrected cluster significance threshold of *p* < .05 based on the Theory of Gaussian Random Fields (Poline et al., 1997, Worsley, 2001). Statistical maps of all analyses were projected onto a standard MNI brain. Group activation maps were visualized using MRIcroGL version 14.3.1 (https://www.nitrc.org/projects/mricrogl/).

To test the interaction between condition (threat or protection) and presentation order (first or second) directly, we ran a separate GLM with four regressors: (1) Condition, (2) Order, (3) Interaction, and (4) Intercept, while keeping all other modeling steps identical to those above.

#### 2.5.3 Post-hoc ROI and Connectivity Analyses

Exploratory analyses were conducted to further test how the hippocampus is functionally connected with cortical circuitry shown to be implicated in safety evaluation (Meyer et al., 2019, Tashjian et al., 2025). These analyses include ROI analyses using a left-ventral hippocampus mask defined from a probabilistic medial temporal lobe atlas independent from the current fMRI data (Fig. 4a; Hindy and Turk-Browne, 2016; Meyer et al., 2019). The standard-space mask was transformed into subject space using FLIRT prior to extraction. For each participant, the mean activation for the Protection First > Threat First contrast was extracted within the left-ventral hippocampus ROI using FSLMEANTS. ROI activations were further analyzed in R using linear regressions to examine associations with age and accuracy. To aid interpretability, we additionally conducted age analyses using a median split, without forcing equal groups, to illustrate differences between younger and older adolescents.

A generalized psychophysiological interaction analysis (gPPI; Friston et al., 1997) was also performed to investigate task-related modulation of functional connectivity patterns between the hippocampus and other brain regions identified in prior work as relevant for safety processing, specifically the anterior vmPFC and ACC (Meyer et al., 2019, Tashjian et al., 2025). The same left-ventral hippocampus mask from the ROI analyses was used. The standard-space mask was transformed into subject space using FLIRT, and the average time series of all voxels within the mask was extracted using FSLMEANTS. The product between the hippocampus time series (physical regressors) and individual regressors for each condition (psychological regressors) was included in each participant’s first-level GLM design matrix. The physical regressors were demeaned, and the psychological regressors were zero-centered. Functional connectivity estimates for the anterior vmPFC (“frontal medial cortex”) and the caudodorsal subregion of the ACC were then extracted from the Protection First condition. These regions were selected based on prior findings of safety circuitry in adults (Meyer et al., 2019, Tashjian et al., 2025). Anterior vmPFC and ACC masks were defined according to the Harvard-Oxford cortical and subcortical structural atlases, independent from the current fMRI data (Fig. 4a). Similar to the ROI analyses, functional connectivity estimates were analyzed in R to examine associations with age (both as continuous and with a median split) and accuracy.

## 3 Results

### 3.1 Behavioral Results

**Fig. 3:**
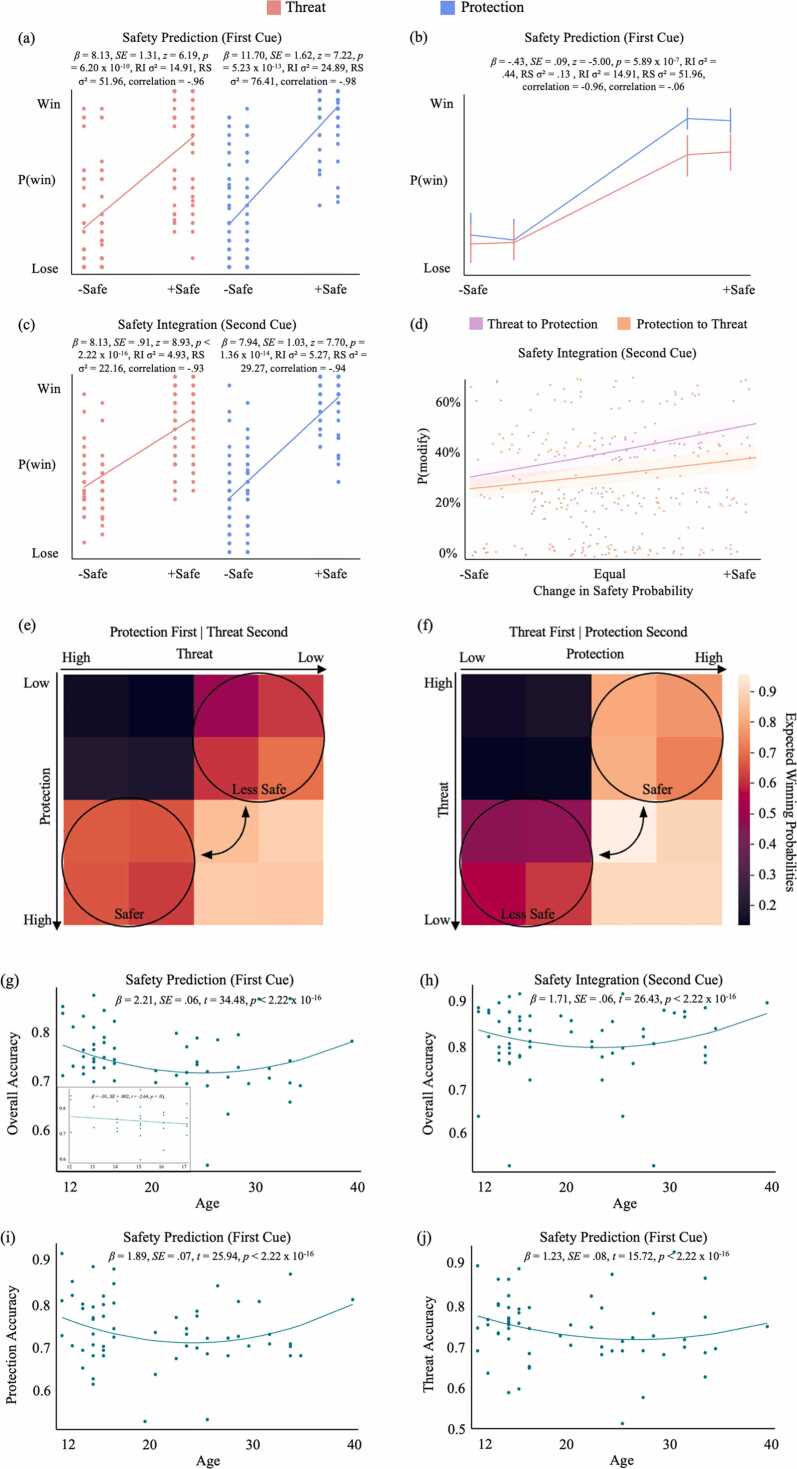
(a, c) Mixed-effects logistic regressions showing the relationship between experimentally established safety probabilities and participants’ safety estimations. Results suggested that Participants were able to track safety probabilities successfully for the first (a) and second (c) cue presentations. (b) For safety prediction, participants estimated a higher probability of winning when protection was presented as the first cue. Vertical error bars represent 95% confidence intervals across participants. (d) For safety integration, participants were more likely to update their safety evaluations when threat cues were followed by protection cues and when safety probabilities were increased. (e-f) Expected safety probabilities. Participants had higher safety estimates when they had more protective weapons and were faced with more threatening animals, compared to when they had less protective weapons and were faced with less threatening animals, regardless of the order of presentation. (g-j) Linear and quadratic regressions showing the relationship between task accuracy and age. Quadratic associations were observed between age and the overall accuracy for safety prediction (g) and safety integration (h) in the full sample, with accuracy decreasing during adolescence and increasing from the mid-twenties. The inset plot in (g) further shows that age was negatively associated with participants’ overall accuracy for safety prediction when considering the adolescent sample only. For safety prediction, quadratic associations were also observed between age and the accuracy of both protection (i) and threat (j), with accuracy decreasing during adolescence and increasing from the mid-twenties. All results remain significant after removing outliers at + /- 2 SD from the mean. RI = random intercept, RS = random slope, correlation = intercept-slope correlation.

Participants estimated a greater probability of winning when exposed to cues with higher experimentally predetermined safety probabilities (Figs. 3a, 3c), indicating that they successfully tracked the task contingencies. For safety prediction (response to the first cue presentation), the source of information affected safety estimations. Participants estimated a higher probability of winning in response to protection cues compared to threat cues (Fig. 3b). Across all models, random effects indicated that participants varied both in their overall baseline tendency to choose “win” and in the extent to which objective probability and cue type influenced responding. Participants also integrated information from both cues (response to the second cue presentation) differently based on the order of presentation and the changes in safety probability (Fig. 3d). Specifically, participants were more likely to update their safety estimations when threat cues were presented first followed by protection cues, and more likely to stick to their initial decisions in the opposite conditions (*β* = .36, *SE* = .12, *z* = 3.04, *p* = .002, random intercept σ² = .25, random slope σ² =.33, intercept-slope correlation = −.53). For example, if participants first saw a lion, they were more likely to revise their safety estimations when receiving a grenade as their weapon. Conversely, participants were more likely to keep their safety estimations the same if they first received the grenade and subsequently saw they were battling a lion. Participants were also more likely to update their estimations when safety probabilities increased from the first cue presentation to the second cue presentation, compared to when safety probabilities decreased (*β* = 1.40, *SE* = .33, *z* = 4.21, *p* = 2.51 ×10^−5^, random intercept σ² = .22, random slope σ² = 3.14, intercept-slope correlation = −.48). In these two models, random effects again indicated that participants differed both in their overall baseline tendency to update their responses and in the extent to which stimulus order and the change in safety probability affected responding. Together, these results indicate that, compared to threat cues, protection cues were more influential for both initial safety predictions and subsequent safety integrations.

When considering animal-weapon pairings (Fig. 3e-f), participants rated their safety as higher for trials involving more protective weapons and more threatening animals, compared to trials involving less protective weapons and less threatening animals, despite equivalent experimentally established safety probabilities (*t*(32) = 2.84, *p* = .008). For all four scenarios with different protection and threat levels, the linear mixed-effects model showed a significant interaction between protection and threat level (*β* = .23, *SE* = .07, *t* = 3.52, *p* = .001), indicating that participants showed the greatest positive safety bias in the “high protection and high threat” scenario.

In our adolescent sample, age was negatively associated with overall accuracy during safety prediction (Fig. 3 g). When adding the adult sample, a quadratic association was observed between age and overall accuracy (Fig. 3 g). Younger adolescents and adults tended to make more accurate safety estimations than older adolescents. To further interrogate this effect, we separately examined safety prediction accuracy (response to the first cue presentation) for protection and threat. Similar age-related patterns were observed for both stimulus types in the full sample (Fig. 3i-j). Age was not significantly associated with safety integration accuracy (response to the second cue presentation) in the adolescent sample (*β* = −.001, *SE* =.003, *z* = -.47, *p* = .64). However, a quadratic association was observed in the full sample (Fig. 3 h). All analyses were robust to outlier removal (+/- 2 SD from the mean), so results are reported for the complete dataset.

### 3.2 Parametric Modulation fMRI Results

**Fig. 4:**
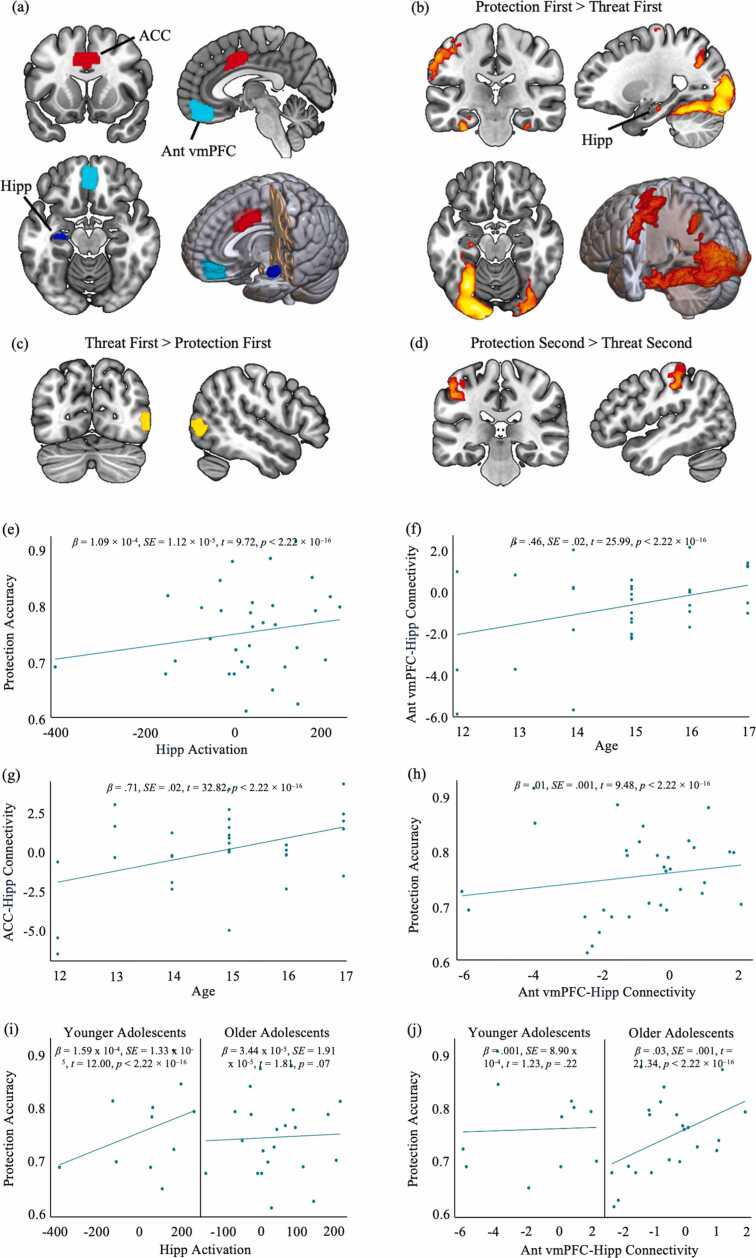
(a) ROIs used in activation and generalized psychophysiological interaction (gPPI) analyses. A ventral hippocampus mask was defined from a probabilistic atlas of the medial temporal lobe; anterior vmPFC and ACC masks were defined according to the Harvard-Oxford cortical and subcortical structural atlases. All masks were independent from the current fMRI data. (b-d) Visualization of significant activation for the contrasts of Protection First > Threat First, Threat First > Protection First, and Protection Second > Threat Second. FLAME1, *Z* > 3.1, FWE-corrected *p* < .05. (e-h) Results from separate main-effects-only linear regression models examining the associations between hippocampal measures and age or protection estimation accuracy. (e) When evaluating protection compared to threat as the first cue, protection estimation accuracy increased as hippocampal activation increased. (f-g) Hippocampal-ACC and hippocampal-anterior vmPFC connectivity increased with age for the Protection First > Threat First contrast. (h) Protection estimation accuracy increased as hippocampal-anterior vmPFC connectivity increased for the Protection First > Threat First contrast. (i-j) After using a median split to divide adolescents into younger (12–14 years) and older (15–17 years) subgroups, we found that hippocampal activation was positively associated with participants’ accuracy for the Protection First > Threat First contrast in younger but not older adolescents. Hippocampus-vmPFC connectivity was positively associated with participants’ accuracy for the Protection First > Threat First contrast in older but not younger adolescents. All results remain significant after removing outliers at + /- 2 SD from the mean. ACC = anterior cingulate cortex; Ant vmPFC = anterior ventromedial prefrontal cortex; Hipp = ventral hippocampus.

We examined neural response to the first and second cue presentations separately, each parametrically modulated to examine neural activation that increased as safety probability increased. The first cue presentation represented the cleanest comparison for the difference in threat versus protection because no information about the corresponding cue was yet available and the safety continuums were identical. When presented first, protection compared to threat evoked greater parametric activation than threat in bilateral visual processing regions (the temporal occipital fusiform cortex, occipital fusiform gyrus, lateral occipital cortex, and occipital pole), and left motor sensory cortices (precentral and postcentral gyri; Fig. 4b). Protection compared to threat also evoked greater parametric activation in the left hippocampus. When presented first, threat compared to protection evoked greater activation in the right lateral occipital cortex (Fig. 4c). The second cue presentation represented an integration phase where the initial estimate for the first cue was updated with information about the second cue (see Fig. 2b). When presented second, protection compared to threat elicited greater parametric activation in visual processing regions (the left temporal occipital fusiform cortex, left occipital fusiform gyrus, left lateral occipital cortex, and bilateral occipital pole) and the left postcentral gyrus (Fig. 4d). No voxels survived cluster correction at *Z* > 3.1, *p* < .05 for threat compared to protection as the second cue. Significant clusters are provided in the Supplementary Materials (Table S1). No significant clusters survived multiple comparison correction at the whole brain level for the interaction between cue type (protection versus threat) and presentation order (first versus second).

### 3.3 Post-hoc ROI and Connectivity Results

GLM results indicated increased left hippocampal activation for protection compared to threat as the first cue (Fig. 4b). To further probe whether this activation was associated with age and accuracy, we conducted ROI analyses by extracting activation from the hippocampus ROI. We estimated two separate main effect models examining the associations between hippocampal activation and age, and between hippocampal activation and participants’ accuracy in estimating the safety probability of protection cues. Hippocampal activation was not significantly associated with age (*β* = −3.90, *SE* = 15.67, *t* = -.25, *p* = .81). Hippocampal activation was positively associated with participants’ protection estimation accuracy (Fig. 4e).

Building on prior work implicating frontal circuitry in safety evaluation (Meyer et al., 2019, Tashjian et al., 2025), we conducted gPPI analyses targeting the hippocampus-anterior vmPFC and hippocampus-ACC pathways. Similar to the ROI analyses, we estimated separate main effect models examining the associations between connectivity and age, and between connectivity and participants’ accuracy in estimating the safety probability of protection. Connectivity for both ROI pairs was positively associated with continuous age in the adolescent sample (12–17 years; Fig. 4f-g). A positive association was observed between hippocampal-anterior vmPFC connectivity and protection estimation accuracy (Fig. 4h). Hippocampal-ACC connectivity was not associated with protection estimation accuracy and was therefore excluded from further analyses (*β* = 1.53 ×10^−4^, *SE* = 5.91 ×10^−4^, *t* = .26, *p* = .80).

We next examined whether age moderated these associations by fitting two additional linear regressions beyond the main effect models reported above: one predicting protection accuracy from hippocampal activation, age, and their interaction, and one predicting protection accuracy from hippocampal-anterior vmPFC connectivity, age, and their interaction. The hippocampal activation model was significant, *F* = 54.59, *p* < 2.22 × 10^−16^. There was a significant interaction between hippocampal activation and continuous age, *β* = −8.15 × 10^−5^, *SE* = 1.03 × 10^−5^, *t* = −7.92, *p* = 3.59 × 10^−15^, indicating that the positive association between hippocampal activation and protection accuracy decreased with age. The hippocampal-anterior vmPFC connectivity model was also significant, *F* = 158.70, *p* < 2.22 × 10^−16^. There was a significant interaction between hippocampal-anterior vmPFC connectivity and continuous age, *β* = .01, *SE* = .0005, *t* = 18.22, *p* < 2.22 × 10^−16^, indicating that the association between hippocampal-anterior vmPFC connectivity and protection accuracy became more positive with increased age. To aid interpretability, we additionally illustrate the effects using a median split at 15 years, with all 15-year-olds included in the older group (younger adolescents: 12–14 years, *N* = 11, 8 females, 73% of the sample, *M*_*Accuracy*_ = 76.31%; older adolescents: 15–17 years, *N* = 22, 11 females, 50% of the sample, *M*_*Accuracy*_ = 74.75%). We found that hippocampal activation was positively associated with participants’ accuracy in estimating the safety probability of protection in younger but not older adolescents (Fig. 4i). In contrast, hippocampus-vmPFC connectivity was positively associated with participants’ protection accuracy in older but not younger adolescents (Fig. 4j). All analyses were robust to outlier removal (+/- 2 SD from the mean), so results are reported for the complete dataset.

## 4 Discussion

The current study examined neural circuits supporting safety evaluation during adolescence. We found that compared to threat, protection recruited distinct neural circuits, including the hippocampus and regions involved in visual and sensorimotor processing. We found evidence that threat and protection cues contributed differently to adolescents’ safety estimations, with protection having more influence. We also found that adolescents’ safety estimations varied with stimulus safety probability and stimulus order. Contrary to our prediction, older adolescents showed less accurate safety estimation than both younger adolescents and adults. Our neuroimaging results further indicated that younger adolescents (12–14 years) showed greater hippocampal engagement when accurately estimating the safety of protection. In older adolescents (15–17 years), connectivity between the hippocampus and anterior vmPFC was associated with more accurate estimation of the safety of protection. These findings suggest age-related differences in the neural mechanisms supporting protection processing, with potential relevance for adolescent behavior associated with safety estimation, including altered risk-taking and vulnerability to anxiety (Blakemore, 2018, Casey et al., 2008, Galván and Rahdar, 2013, Zacharek et al., 2021).

We extended previous adult work on protection-based safety processing (Tashjian et al., 2025) to adolescents using the Safety Evaluation Task, which examined the dynamic interplay between threat and protection cues. Our design shares key features with traditional fear conditioning paradigms, in particular conditioned inhibition and differential conditioning. When presented in isolation during the first half of each trial, threat and protection cues are most closely analogous to CS + and CS- in differential conditioning, each predicting a different outcome. In trials where a threat cue is immediately followed by a protection cue, protection serves as a partial conditioned inhibitor, signaling a reduced probability of an aversive outcome. While grounded in the fear conditioning framework, our design extends it by implementing probabilistic cue-outcome contingencies to modulate safety. We treat protection cues as independent entities, in addition to their threat-inhibitory role. This approach better approximates contexts where threat and protection co-occur and carry their own inherent value, which provides a more nuanced understanding of adolescent safety processing. Moreover, the cues presented in this study were recognizable and had inherent value (e.g., a cat is less dangerous than a lion) that reduced the need to learn, differing from most classical conditioning paradigms that use neutral, unfamiliar cues.

Our behavioral results suggest that adolescents’ safety estimations varied with stimulus type, stimulus order, and stimulus safety probability. During initial safety evaluations (first cue presentation), adolescents were more likely to predict a win following protection compared to threat. When comparing two integration scenarios with the same overall objective winning probability, adolescents estimated a higher probability of winning in the “high protection and high threat scenario” than in the “low protection and low threat” scenario, rather than rating them as equally safe. Taken together, these results suggest that adolescents’ safety estimations are more strongly influenced by protection than by threat, supporting our framework that protection is an important component of safety estimation in contexts involving physical danger. The order of cue presentation had an impact on safety updating: Adolescents were more likely to revise their safety estimations when cues were presented in a threat-to-protection sequence, and more likely to maintain their initial decision in the opposite condition, consistent with our finding that protection exerted a stronger influence on adolescents’ safety estimations. Finally, our behavioral results suggest that adolescents actively tracked safety probability across the continuum, reporting higher perceived safety for both protection and threat stimuli associated with higher winning probabilities. Interestingly, changes in safety probability showed an asymmetric effect on adolescents’ safety updating, such that they were more likely to revise their safety estimations when safety probability increased than when it decreased. These findings suggest that rather than purely responding to stimulus type, adolescents considered stimulus winning probability when completing the task. The behavioral patterns we found in our adolescent sample are broadly consistent with prior work using a similar task in a separate sample of adults (Tashjian et al., 2025). Both adolescents and adults estimated greater safety when the first-presented stimulus was protection, were more likely to update safety estimates when protection followed threat, and were sensitive to stimulus safety probability, particularly when safety increased. The two samples also showed some behavioral differences. Adults were generally more confident and showed less differentiation in their safety estimations across threat-protection conditions. Moreover, the adult bias to rate the “high protection and high threat” scenario as safer emerged only when threat was presented first.

We observed age-related differences in protection estimation accuracy, with linear decreases in our adolescent sample and quadratic effects when combining the adolescent and adult samples. The U-shaped pattern may suggest that mid-to-late adolescence is a period of vulnerability in accurate protection estimation. Alternatively, the decline could aid specific goals of adolescence, including exploration, by reducing attention to threat. Although recent evidence suggests that extinction learning reaches maturity by adolescence (Abend et al., 2020, Widegren et al., 2025), our study demonstrates that when incorporating protection as a component of safety processing, adolescents show distinct behavioral patterns. Our neuroimaging results converged with our behavioral findings, showing age-associated differences in mechanisms underlying accurate protection estimation. For younger adolescents, protection accuracy was positively associated with hippocampal activation but not with connectivity between the hippocampus and ACC or vmPFC. Older adolescents with higher hippocampal-anterior vmPFC connectivity demonstrated more accurate safety estimations for protection, while hippocampal activation alone was no longer associated with performance.

Our results highlight differences in neural mechanisms supporting the evaluation of protection across the adolescent period, consistent with prior work on adolescent cognition and safety processing. In our study, younger adolescents predominantly engaged the hippocampus when evaluating protection, which may reflect compensation for still-developing prefrontal systems through greater reliance on hippocampal systems associated with more local, memory-based processing. The hippocampus may facilitate memory retrieval about protection-relevant information before integrated hippocampal-prefrontal circuits take over these functions later in development. Importantly, Sastre et al. (2016) demonstrated that hippocampal activation during episodic retrieval is higher in children and decreases with age, which dovetails with prefrontal regions becoming more engaged to exert inhibitory control over the hippocampus, following a similar age-related pattern to what we observed during protection evaluation.

Instead of engaging primarily in hippocampal processing, we found that older adolescents recruited a more integrated circuit involving the vmPFC to accurately evaluate protection. This pattern is consistent with previous research showing progressive strengthening of long-range functional connections between the hippocampus and prefrontal cortex throughout adolescence (Calabro et al., 2020, Murty et al., 2016). The development of these integrated circuits facilitates the retrieval and incorporation of relevant prior experiences and the formation of sophisticated schemas, both informing optimal behavioral responses to novel situations (Gluth et al., 2015, Spalding et al., 2015, van Kesteren et al., 2010, Voss et al., 2015). The importance of this circuitry has been demonstrated in adults, with Tashjian et al. (2025) showing that the anterior vmPFC is specifically involved in processing protection cues. Similarly, Milad et al. (2008) found that successful recall of extinction memory activates the vmPFC and hippocampus in concert, and Harrison et al. (2017) identified the vmPFC as critical for processing safety signals in differential fear conditioning. Moreover, greater functional connectivity between the hippocampus and vmPFC was observed for stimuli that were less similar to the CS + during fear generalization, likely reflecting the hippocampus’s role in supporting vmPFC-mediated fear inhibition (Lissek et al., 2014). In our task, greater reliance on hippocampal-vmPFC connectivity in older adolescents may reflect increasing capacity to adeptly use available resources with age.

One interpretation of our behavioral and neuroimaging results is that older adolescents are going through a developmental transition, moving away from established hippocampal mechanisms before fully developing more sophisticated hippocampal-prefrontal circuits for protection evaluation. The hippocampus stores episodic memories and contextual information, while the prefrontal cortex provides executive control, planning, and decision-making capabilities. Their connectivity allows past experiences (stored in the hippocampus) to directly inform complex decision-making processes (mediated by the PFC; Preston and Eichenbaum, 2013; Murty et al., 2016). However, developing hippocampal-prefrontal circuits requires greater cognitive resources and coordination than either mature integrated networks or established single-region processing. Therefore, unlike younger adolescents who effectively use hippocampal processing alone or adults with fully developed hippocampal-prefrontal connectivity, older adolescents may experience a temporary functional gap. It is important to note that the current study used a cross-sectional design, which prevents causal claims about within-person developmental transitions or the timing of any changes in neural circuitry. Moreover, given that our task required multidimensional information integration, these age-related differences may partly reflect broader developmental changes in integration ability, rather than being specific to safety processing.

Although hippocampal-ACC connectivity was positively associated with age, similar to hippocampal-vmPFC connectivity, only the latter was linked to protection accuracy in our data. This may appear to contradict Meyer et al. (2019), who found that hippocampus-ACC connectivity, but not hippocampus-anterior vmPFC connectivity, was involved in threat inhibition via safety signals. However, we interpret our findings as extending rather than contradicting those of Meyer and colleagues. Specifically, hippocampus-anterior vmPFC connectivity is likely involved in safety processing when protection is perceived as independent from threat, whereas hippocampus-ACC connectivity might be more relevant for safety signals that directly inhibit threat, which conceptually involves more switching from threat to safe associations (Jovanovic et al., 2013). This proposed distinction between neural circuits may have important implications for clinical interventions. Many current psychotherapies for anxiety disorders, such as exposure-based cognitive-behavioral therapy, are grounded in fear conditioning paradigms addressing how neutral cues acquire threat value and how fear can persist or generalize (Craske et al., 2014, Ryan et al., 2019). However, these methods are found to be ineffective for approximately 50% of patients (Ginsburg et al., 2014, Springer et al., 2018). One possibility is that these patients have an anxiety symptom profile in which threat responding is appropriate but the integration of protection resources is challenging. For these patients, therapeutic approaches that focus on protective resources may offer a supplementary approach to improve treatment outcomes through complementary mechanisms.

Results from the current study also provide insight into how competition between threat and protection cues is resolved within the visual cortex, showing greater activation in response to protection. Previous evidence suggests that the ventral visual networks play pivotal roles in tracking stimuli that are salient or highly relevant to behavior (Lang and Bradley, 2010, Wang et al., 2022) and supporting memory retrieval for the stimulus itself or similar experiences (Hofstetter et al., 2012). Among the most salient of all stimuli is threat, to which the sensory system exhibits biases for survival (Damaraju et al., 2009, Padmala and Pessoa, 2008, Stolarova et al., 2006). This preference for threat persists with the co-occurrence of threat and safety signals in extinction and conditioned inhibition (Miskovic and Keil, 2013, Talmi et al., 2019). However, and contradicting previous studies, we found preferential processing of the visual system for protection compared to threat, which may be attributed to different constructs of safety. In the conditioning framework, a threat-safety competition is evoked during safety acquisition, wherein the presence of a safety cue eliminates the significance of the threat cue. In the current study, safety is instead conferred in the presence of threat through protection. Protection cues more closely resemble threat cues that are linked to sensory amplification: They are highly relevant and salient on their own, rather than having meaning for the relevance of an independent threat cue. In this case, sensory systems respond with increased activation to protection. This pattern aligns with predictive coding frameworks, suggesting that when protection cues carry high informational value for survival outcomes, the visual cortex prioritizes their processing to optimize perceptual decision-making, contrasting with traditional threat-biased attention allocation (Mobbs et al., 2015, Price and Gavornik, 2022). The distinction between previous studies and the current one shows that actively increasing safety through positive protection cues may facilitate engagement of lower-tier sensory cortices, optimizing safety perception and accurate decision-making.

Several limitations should be considered when interpreting our findings. First, larger samples would provide greater opportunities for examining individual differences, and longitudinal inquiries would support the interrogation of developmental trajectories. Our paradigm focused on binary win/lose choices rather than continuous ratings. Continuous ratings may have captured more subtle variations in safety estimation, but also run the risk of a midpoint bias common to uncertain and probabilistic tasks. We used a fictitious world (battles with animals and weapons), which reduces ecological validity. Our study did not collect skin conductance response (SCR), which is commonly included as a physiological measure in fear conditioning. However, SCR is poorly suited to fMRI, given that supine posture and the cold MRI environment attenuate SCR and signal quality (Morriss et al., 2019). Thus, the current study relied on behavioral responses to confirm task-relevant responding. This limits comparison with prior studies that rely on SCR to validate successful conditioning without corresponding behavioral measures. We used real-world threat and protection cues, presenting stimuli that are both perceptually threatening and modifying their safety probability across matching probability spectrums. However, examining how different types of protection cues (e.g., social versus non-social) engage these neural systems across development would provide further insights into adolescent safety processing.

Our study provides novel insights into the complex neurodevelopmental processes underlying safety processing during adolescence. Our findings suggest that protection is a distinct and influential component of safety estimation in contexts involving physical danger. Our findings also reveal age-related differences in the neural mechanisms supporting protection evaluation during adolescence. Accurate safety estimation for protection was linked to hippocampal processing in younger adolescents but integrated hippocampal-prefrontal processing in older adolescents. The visual cortex was more engaged for protection compared to threat evaluation, suggesting a role in facilitating safety processing for protection information. These findings have implications for understanding adolescent risk-taking behavior and vulnerability to anxiety, potentially guiding the development of interventions aimed at strengthening the ability to recognize protection during this critical developmental period. Interventions may be most effective when tailored to adolescents’ developmental stage, with younger adolescents potentially benefiting from memory-based approaches that leverage hippocampal processing, while older adolescents may require interventions that support the maturation of integrated prefrontal-subcortical circuits.

## CRediT authorship contribution statement

**Marta I. Garrido:** Writing – review & editing, Supervision. **Sarah M. Tashjian:** Writing – review & editing, Visualization, Validation, Supervision, Software, Resources, Project administration, Methodology, Funding acquisition, Formal analysis, Data curation, Conceptualization. **Yubing Zhang:** Writing – review & editing, Writing – original draft, Visualization, Software, Methodology, Investigation, Formal analysis, Data curation, Conceptualization. **Madeline K. Coates:** Writing – review & editing, Investigation.

## Funding sources

This work was supported by a National Health and Medical Research Council (NHMRC) Investigator Grant (2033400) and a Brain and Behavior Research Foundation Young Investigator Grant to SMT (30788). The funders played no role in study design, data collection or analysis, decision to publish, or preparation of the manuscript.

## Declaration of Competing Interest

The authors declare that they have no known competing financial interests or personal relationships that could have appeared to influence the work reported in this paper.

## Data Availability

Task code and behavioral data are available through the Open Science Framework (OSF; https://doi.org/10.17605/OSF.IO/V3KPX). Neuroimaging data are available through the Science Data Bank (https://doi.org/10.57760/sciencedb.26549).

## Glossary

**Conditional discrimination**: a learning paradigm in which the meaning of a cue depends on its combination with other cues. For example, if AX + signals threat and BX- signals the absence of threat, presenting AB yields a reduced fear response compared to AX + , reflecting transfer of safety from B to A.
**Conditioned inhibition**: a learning paradigm in which a neutral cue predicts the absence of an aversive outcome when paired with a threat cue. For example, if stimulus A alone signals threat but AX- signals the absence of threat, X inhibits the fear response to A.
**Danger**: the objective potential for harm or adverse outcome that exists in a situation.
**Differential fear conditioning**: a learning paradigm in which one stimulus (CS+) is paired with an aversive outcome (US) while another (CS-) is not, leading to the CS + eliciting increased fear/threat response compared to CS-.
**Extinction**: the process by which a conditioned fear/threat response decreases when the CS + is repeatedly presented without the US.
**Protection**: resources available to an agent, ranging from defensive tools to cognitive capabilities, that increase safety. Self-oriented (e.g., aligned with the agent rather than the threat).
**Safety**: the state or probability of being free from harm or adverse outcome.
**Safety estimation**: the cognitive process of calculating safety probability (‘how safe am I?’).
**Safety evaluation**: the comprehensive process of estimating one’s safety through detection, prediction, and integration to guide behavioral responses.
**Safety integration**: the process of combining multiple elements (e.g., threat and protection) to form a comprehensive safety estimation.
**Safety prediction**: the process of estimating safety based on partial information, specifically when only one element (e.g., threat or protection, but not both) is known.
**Safety processing**: the broader neural and cognitive mechanisms involved in handling safety information, including detection, prediction, integration, evaluation, and decision-making.
**Threat**: external cues that signal potential danger to the agent evaluating them.
**Threat/Safety detection**: the basic perceptual process of identifying threat/safety cues in the environment.

## References

[bib1] Abend R., Gold A.L., Britton J.C., Michalska K.J., Shechner T., Sachs J.F., Winkler A.M., Leibenluft E., Averbeck B.B., Pine D.S. (2020). Anticipatory threat responding: Associations with anxiety, development, and brain structure. Biol. Psychiatry.

[bib2] Anderson P.L., Price M., Edwards S.M., Obasaju M.A., Schmertz S.K., Zimand E., Calamaras M.R. (2013). Virtual reality exposure therapy for social anxiety disorder: A randomized controlled trial. J. Consult. Clin. Psychol..

[bib3] Bach D.R., Dayan P. (2017). Algorithms for survival: A comparative perspective on emotions. Nat. Rev. Neurosci..

[bib4] Battaglia S., Harrison B.J., Fullana M.A. (2022). Does the human ventromedial prefrontal cortex support fear learning, fear extinction or both? A commentary on subregional contributions. Mol. Psychiatry.

[bib5] Beckmann C.F., Jenkinson M., Smith S.M. (2003). General multilevel linear modeling for group analysis in fMRI. NeuroImage.

[bib6] Beidel D.C., Alfano C.A., Kofler M.J., Rao P.A., Scharfstein L., Sarver N.W. (2014). The impact of social skills training for social anxiety disorder: A randomized controlled trial. J. Anxiety Disord..

[bib7] Blakemore S.J. (2018). Avoiding social risk in adolescence. Curr. Dir. Psychol. Sci..

[bib8] Britton J.C., Grillon C., Lissek S., Norcross M.A., Szuhany K.L., Chen G., Ernst M., Nelson E.E., Leibenluft E., Shechner T., Pine D.S. (2013). Response to learned threat: An fMRI study in adolescent and adult anxiety. Am. J. Psychiatry.

[bib9] Calabro F.J., Murty V.P., Jalbrzikowski M., Tervo-Clemmens B., Luna B. (2020). Development of hippocampal-prefrontal cortex interactions through adolescence. Cereb. Cortex.

[bib10] Casey B.J., Galván A., Somerville L.H. (2016). Beyond simple models of adolescence to an integrated circuit-based account: A commentary. Dev. Cogn. Neurosci..

[bib11] Casey B.J., Getz S., Galvan A. (2008). The adolescent brain. Dev. Rev..

[bib12] Ciranka S., van den Bos W. (2021). Adolescent risk-taking in the context of exploration and social influence. Dev. Rev..

[bib13] Craske M.G., Treanor M., Conway C.C., Zbozinek T., Vervliet B. (2014). Maximizing exposure therapy: An inhibitory learning approach. Behav. Res. Ther..

[bib14] Crone E.A., Dahl R.E. (2012). Understanding adolescence as a period of social-affective engagement and goal flexibility. Nat. Rev. Neurosci..

[bib15] Damaraju E., Huang Y.M., Barrett L.F., Pessoa L. (2009). Affective learning enhances activity and functional connectivity in early visual cortex. Neuropsychologia.

[bib16] Dumontheil I. (2014). Development of abstract thinking during childhood and adolescence: The role of rostrolateral prefrontal cortex. Dev. Cogn. Neurosci..

[bib17] Edelson S.M., Reyna V.F. (2021). How fuzzy-trace theory predicts development of risky decision making, with novel extensions to culture and reward sensitivity. Dev. Rev..

[bib18] Esteban O., Markiewicz C.J., Blair R.W., Moodie C.A., Isik A.I., Erramuzpe A., Kent J.D., Goncalves M., DuPre E., Snyder M., Oya H., Ghosh S.S., Wright J., Durnez J., Poldrack R.A., Gorgolewski K.J. (2019). fMRIPrep: A robust preprocessing pipeline for functional MRI. Nat. Methods.

[bib19] Friston K.J., Buechel C., Fink G.R., Morris J., Rolls E., Dolan R.J. (1997). Psychophysiological and modulatory interactions in neuroimaging. NeuroImage.

[bib20] Fullana M.A., Dunsmoor J.E., Schruers K.R., Savage H.S., Bach D.R., Harrison B.J. (2020). Human fear conditioning: From neuroscience to the clinic. Behav. Res. Ther..

[bib21] Fuster J.M. (2002). Frontal lobe and cognitive development. J. Neurocytol..

[bib22] Galván A., Rahdar A. (2013). The neurobiological effects of stress on adolescent decision making. Neuroscience.

[bib23] Ganella D.E., Drummond K.D., Ganella E.P., Whittle S., Kim J.H. (2018). Extinction of conditioned fear in adolescents and adults: A human fMRI study. Front. Human. Neurosci..

[bib24] Ginsburg G.S., Becker E.M., Keeton C.P., Sakolsky D., Piacentini J., Albano A.M., Compton S.N., Lyengar S., Sullivan K., Caporino N., Peris T., Birmaher B., Rynn M., March J., Kendall P.C. (2014). Naturalistic follow-up of youths treated for pediatric anxiety disorders. JAMA Psychiatry.

[bib25] Gluth S., Sommer T., Rieskamp J., Büchel C. (2015). Effective connectivity between hippocampus and ventromedial prefrontal cortex controls preferential choices from memory. Neuron.

[bib26] Gold A.L., Abend R., Britton J.C., Behrens B., Farber M., Ronkin E., Chen G., Leibenluft E., Pine D.S. (2020). Age differences in the neural correlates of anxiety disorders: An fMRI study of response to learned threat. Am. J. Psychiatry.

[bib27] Gopnik A., O’Grady S., Lucas C.G., Griffiths T.L., Wente A., Bridgers S., Aboody R., Fung H., Dahl R.E. (2017). Changes in cognitive flexibility and hypothesis search across human life history from childhood to adolescence to adulthood. Proc. Natl. Acad. Sci..

[bib28] Grasser L.R., Jovanovic T. (2021). Safety learning during development: Implications for development of psychopathology. Behav. Brain Res..

[bib29] Greco J.A., Liberzon I. (2016). Neuroimaging of fear-associated learning. Neuropsychopharmacology.

[bib30] Harrewijn A., Kitt E.R., Abend R., Matsumoto C., Odriozola P., Winkler A.M., Leibenluft E., Pine D.S., Gee D.G. (2021). Comparing neural correlates of conditioned inhibition between children with and without anxiety disorders—A preliminary study. Behav. Brain Res..

[bib31] Harrison B.J., Fullana M.A., Via E., Soriano-Mas C., Vervliet B., Martínez-Zalacaín I., Pujol J., Davey C.G., Kircher T., Straube B., Cardoner N. (2017). Human ventromedial prefrontal cortex and the positive affective processing of safety signals. NeuroImage.

[bib32] Hartley C.A., Somerville L.H. (2015). The neuroscience of adolescent decision-making. Curr. Opin. Behav. Sci..

[bib33] Hindy N.C., Turk-Browne N.B. (2016). Action-based learning of multistate objects in the medial temporal lobe. Cereb. Cortex.

[bib34] Hofstetter C., Achaibou A., Vuilleumier P. (2012). Reactivation of visual cortex during memory retrieval: content specificity and emotional modulation. NeuroImage.

[bib35] Jenkinson M., Beckmann C.F., Behrens T.E., Woolrich M.W., Smith S.M. (2012). Fsl. NeuroImage.

[bib36] Jovanovic T., Kazama A., Bachevalier J., Davis M. (2013). Impaired safety signal learning may be a biomarker of PTSD. Neuropharmacology.

[bib37] van Kesteren M.T., Fernández G., Norris D.G., Hermans E.J. (2010). Persistent schema-dependent hippocampal-neocortical connectivity during memory encoding and postencoding rest in humans. Proc. Natl. Acad. Sci..

[bib38] Kong E., Monje F.J., Hirsch J., Pollak D.D. (2014). Learning not to fear: Neural correlates of learned safety. Neuropsychopharmacology.

[bib39] Kveraga K., Ghuman A.S., Bar M. (2007). Top-down predictions in the cognitive brain. Brain Cogn..

[bib40] Laing P.A., Felmingham K.L., Davey C.G., Harrison B.J. (2022). The neurobiology of Pavlovian safety learning: Towards an acquisition-expression framework. Neurosci. & Biobehav. Rev..

[bib41] Laing P.A., Harrison B.J. (2021). Safety learning and the Pavlovian conditioned inhibition of fear in humans: Current state and future directions. Neurosci. & Biobehav. Rev..

[bib42] Laing P.A., Vervliet B., Fullana M.A., Savage H.S., Davey C.G., Felmingham K.L., Harrison B.J. (2021). Characterizing human safety learning via Pavlovian conditioned inhibition. Behav. Res. Ther..

[bib43] Lang P.J., Bradley M.M. (2010). Emotion and the motivational brain. Biol. Psychol..

[bib44] Larsen B., Luna B. (2018). Adolescence as a neurobiological critical period for the development of higher-order cognition. Neurosci. & Biobehav. Rev..

[bib45] Lau J.Y.F., Britton J.C., Nelson E.E., Angold A., Ernst M., Goldwin M., Grillon C., Leibenluft E., Lissek S., Norcross M., Shiffrin N., Pine D.S. (2011). Distinct neural signatures of threat learning in adolescents and adults. Proc. Natl. Acad. Sci..

[bib46] LeDoux J.E., Pine D.S. (2016). Using neuroscience to help understand fear and anxiety: A two-system framework. Am. J. Psychiatry.

[bib47] Lee F.S., Heimer H., Giedd J.N., Lein E.S., Šestan N., Weinberger D.R., Casey B.J. (2014). Adolescent mental health—Opportunity and obligation. Science.

[bib48] Lissek S., Bradford D.E., Alvarez R.P., Burton P., Espensen-Sturges T., Reynolds R.C., Grillon C. (2014). Neural substrates of classically conditioned fear-generalization in humans: a parametric fMRI study. Social. Cogn. Affect. Neurosci..

[bib49] Luciana M., Conklin H.M., Hooper C.J., Yarger R.S. (2005). The development of nonverbal working memory and executive control processes in adolescents. Child. Dev..

[bib50] Maier S.F., Amat J., Baratta M.V., Paul E., Watkins L.R. (2006). Behavioral control, the medial prefrontal cortex, and resilience. Dialog.-. Clin. Neurosci..

[bib51] Marques J.P., Kober T., Krueger G., van der Zwaag W., van de Moortele P.F., Gruetter R. (2010). MP2RAGE, a self bias-field corrected sequence for improved segmentation and T1-mapping at high field. NeuroImage.

[bib52] Meyer H.C., Odriozola P., Cohodes E.M., Mandell J.D., Li A., Yang R., Hall B.S., Haberman J.T., Zacharek S.J., Liston C., Lee F.S., Gee D.G. (2019). Ventral hippocampus interacts with prelimbic cortex during inhibition of threat response via learned safety in both mice and humans. Proc. Natl. Acad. Sci..

[bib53] Micale V., Stepan J., Jurik A., Pamplona F.A., Marsch R., Drago F., Eder M., Wotjak C.T. (2017). Extinction of avoidance behavior by safety learning depends on endocannabinoid signaling in the hippocampus. J. Psychiatr. Res..

[bib54] Milad M.R., Orr S.P., Lasko N.B., Chang Y., Rauch S.L., Pitman R.K. (2008). Presence and acquired origin of reduced recall for fear extinction in PTSD: Results of a twin study. J. Psychiatr. Res..

[bib55] Mills K.L., Goddings A.L., Clasen L.S., Giedd J.N., Blakemore S.J. (2014). The developmental mismatch in structural brain maturation during adolescence. Dev. Neurosci..

[bib56] Miskovic V., Keil A. (2013). Perceiving threat in the face of safety: Excitation and inhibition of conditioned fear in human visual cortex. J. Neurosci..

[bib57] Mobbs D., Hagan C.C., Dalgleish T., Silston B., Prévost C. (2015). The ecology of human fear: Survival optimization and the nervous system. Front. Neurosci..

[bib58] Mobbs D., Headley D.B., Ding W., Dayan P. (2020). Space, time, and fear: Survival computations along defensive circuits. Trends Cogn. Sci..

[bib59] Moeller S., Yacoub E., Olman C.A., Auerbach E., Strupp J., Harel N., Uğurbil K. (2010). Multiband multislice GE-EPI at 7 tesla, with 16-fold acceleration using partial parallel imaging with application to high spatial and temporal whole-brain fMRI. Magn. Reson. Med..

[bib60] Morris L.S., Kundu P., Costi S., Collins A., Schneider M., Verma G., Balchandani P., Murrough J.W. (2019). Ultra-high field MRI reveals mood-related circuit disturbances in depression: A comparison between 3-Tesla and 7-Tesla. Transl. Psychiatry.

[bib61] Morriss J., Christakou A., van Reekum C.M. (2019). Multimodal evidence for delayed threat extinction learning in adolescence and young adulthood. Sci. Rep..

[bib62] Mumford J.A., Poline J.B., Poldrack R.A. (2015). Orthogonalization of regressors in fMRI models. PLoS. One.

[bib63] Murty V.P., Calabro F., Luna B. (2016). The role of experience in adolescent cognitive development: Integration of executive, memory, and mesolimbic systems. Neurosci. & Biobehav. Rev..

[bib64] Odriozola P., Gee D.G. (2021). Learning about safety: Conditioned inhibition as a novel approach to fear reduction targeting the developing brain. Am. J. Psychiatry.

[bib65] Padmala S., Pessoa L. (2008). Affective learning enhances visual detection and responses in primary visual cortex. J. Neurosci..

[bib66] Pattwell S.S., Duhoux S., Hartley C.A., Johnson D.C., Jing D., Elliott M.D., Ruberry E.J., Powers A., Mehta N., Yang R.R., Soliman F., Glatt C.E., Casey B.J., Ninan I., Lee F.S. (2012). Altered fear learning across development in both mouse and human. Proc. Natl. Acad. Sci..

[bib67] Phelps E.A., Delgado M.R., Nearing K.I., LeDoux J.E. (2004). Extinction learning in humans: Role of the amygdala and vmPFC. Neuron.

[bib68] Poline J.B., Worsley K.J., Evans A.C., Friston K.J. (1997). Combining spatial extent and peak intensity to test for activations in functional imaging. NeuroImage.

[bib69] Preston A.R., Eichenbaum H. (2013). Interplay of hippocampus and prefrontal cortex in memory. Curr. Biol..

[bib70] Price B.H., Gavornik J.P. (2022). Efficient temporal coding in the early visual system: Existing evidence and future directions. Front. Comput. Neurosci..

[bib71] Qi S., Hassabis D., Sun J., Guo F., Daw N., Mobbs D. (2018). How cognitive and reactive fear circuits optimize escape decisions in humans. Proc. Natl. Acad. Sci..

[bib72] R Core Team, 2021. R A Lang. Environ. Stat. Comput. (Version 4.5.1) [Computer software] https://www.R-project.org/.

[bib73] Ryan K.M., Zimmer-Gembeck M.J., Neumann D.L., Waters A.M. (2019). The need for standards in the design of differential fear conditioning and extinction experiments in youth: A systematic review and recommendations for research on anxiety. Behav. Res. Ther..

[bib74] Sangha S., Diehl M.M., Bergstrom H.C., Drew M.R. (2020). Know safety, no fear. Neurosci. & Biobehav. Rev..

[bib75] Sastre M., Wendelken C., Lee J.K., Bunge S.A., Ghetti S. (2016). Age- and performance-related differences in hippocampal contributions to episodic retrieval. Dev. Cogn. Neurosci..

[bib76] Schiller D., Levy I., Niv Y., LeDoux J.E., Phelps E.A. (2008). From fear to safety and back: Reversal of fear in the human brain. J. Neurosci..

[bib77] Somerville L.H., Sasse S.F., Garrad M.C., Drysdale A.T., Abi Akar N., Insel C., Wilson R.C. (2017). Charting the expansion of strategic exploratory behavior during adolescence. J. Exp. Psychol. General..

[bib78] Spalding K.N., Jones S.H., Duff M.C., Tranel D., Warren D.E. (2015). Investigating the neural correlates of schemas: Ventromedial prefrontal cortex is necessary for normal schematic influence on memory. J. Neurosci..

[bib79] Spear L.P. (2000). The adolescent brain and age-related behavioral manifestations. Neurosci. & Biobehav. Rev..

[bib80] Springer K.S., Levy H.C., Tolin D.F. (2018). Remission in CBT for adult anxiety disorders: A meta-analysis. Clin. Psychol. Rev..

[bib81] Stolarova M., Keil A., Moratti S. (2006). Modulation of the C1 visual event-related component by conditioned stimuli: Evidence for sensory plasticity in early affective perception. Cereb. Cortex.

[bib82] Talmi D., Slapkova M., Wieser M.J. (2019). Testing the possibility of model-based Pavlovian control of attention to threat. J. Cogn. Neurosci..

[bib83] Tashjian S.M., Cussen J., Deng W., Zhang B., Mobbs D. (2025). Subregions in the ventromedial prefrontal cortex integrate threat and protective information to meta-represent safety. PLoS. Biol..

[bib84] Tashjian S.M., Zbozinek T.D., Mobbs D. (2021). A decision architecture for safety computations. Trends Cogn. Sci..

[bib85] Torrisi S., Chen G., Glen D., Bandettini P.A., Baker C.I., Reynolds R., Yen-Ting Liu J., Leshin J., Balderston N., Grillon C., Ernst M. (2018). Statistical power comparisons at 3T and 7T with a GO / NOGO task. NeuroImage.

[bib86] Tovote P., Esposito M.S., Botta P., Chaudun F., Fadok J.P., Markovic M., Wolff S.B.E., Ramakrishnan C., Fenno L., Deisseroth K., Herry C., Arber S., Lüthi A. (2016). Midbrain circuits for defensive behaviour. Nature.

[bib87] Towner E., Chierchia G., Blakemore S.J. (2023). Sensitivity and specificity in affective and social learning in adolescence. Trends Cogn. Sci..

[bib88] Viessmann O., Polimeni J.R. (2021). High-resolution fMRI at 7 Tesla: Challenges, promises and recent developments for individual-focused fMRI studies. Curr. Opin. Behav. Sci..

[bib89] Voss J.L., O’Neil J.T., Kharitonova M., Briggs-Gowan M.J., Wakschlag L.S. (2015). Adolescent development of context-dependent stimulus-reward association memory and its neural correlates. Front. Human. Neurosci..

[bib90] Wang L., Huang L., Li M., Wang X., Wang S., Lin Y., Zhang X. (2022). An awareness-dependent mapping of saliency in the human visual system. NeuroImage.

[bib91] Waters A.M., Theresiana C., Neumann D.L., Craske M.G. (2017). Developmental differences in aversive conditioning, extinction, and reinstatement: A study with children, adolescents, and adults. J. Exp. Child. Psychol..

[bib92] Weil L.G., Fleming S.M., Dumontheil I., Kilford E.J., Weil R.S., Rees G., Dolan R., Blakemore S.J. (2013). The development of metacognitive ability in adolescence. Conscious. Cogn..

[bib93] Wen Z., Pace-Schott E.F., Lazar S.W., Rosén J., Åhs F., Phelps E.A., LeDoux J.E., Milad M.R. (2024). Distributed neural representations of conditioned threat in the human brain. Nat. Commun..

[bib94] Widegren E., Vegelius J., Frick M.A., Roy A.A., Möller S., Kleberg J.L., Hoppe J.M., Hjorth O., Fällmar D., Pine D.S., Brocki K., Gingnell M., Frick A. (2025). Fear extinction retention in children, adolescents, and adults. Dev. Cogn. Neurosci..

[bib95] Worsley K.J., Jezzard P., Matthews P.M., Smith S.M. (2001). Functional MRI: An Introduction to Methods.

[bib96] Wu K., Wang D., Wang Y., Tang P., Li X., Pan Y., Tao H.W., Zhang L.I., Liang F. (2023). Distinct circuits in anterior cingulate cortex encode safety assessment and mediate flexibility of fear reactions. Neuron.

[bib97] Zacharek S.J., Kribakaran S., Kitt E.R., Gee D.G. (2021). Leveraging big data to map neurodevelopmental trajectories in pediatric anxiety. Dev. Cogn. Neurosci..

