# Supplementary material for: Age-related differences in hippocampal network engagement during safety processing in adolescents
Source: Dev Cogn Neurosci. 2026 Jul 28;81:101791. doi: 10.1016/j.dcn.2026.101791 (PMC13452386; doi:10.1016/j.dcn.2026.101791)
Supplement: Supplementary file 1 [file mmc1.docx]

# Supplemental Materials

## Whole-Brain Cluster Reporting

Global cluster peaks for the group-level GLM (contrasts: Protection First > Threat First, Threat First > Protection First, and Protection Second > Threat Second) are reported in Table S1.

**Table S1**

*Significant Clusters from Group-Level GLM*

| Cluster Peak Region | R/L | Voxel Count | *p*-Value | Max *Z*-Value | Peak MNI Coordinates | | |
| --- | --- | --- | --- | --- | --- | --- | --- |
|  |  |  |  |  | X | Y | Z |
| Protection First > Threat First | | | | | | | |
| Occipital Pole | L | 4269 | 3.57 x 10^-23^ | 6.85 | -12 | -98 | -16 |
| Postcentral Gyrus | L | 1266 | 4.75 x 10^-10^ | 5.15 | -58 | -20 | 44 |
| Occipital Pole | R | 1042 | 9.04 x 10^-9^ | 4.68 | 32 | -90 | -20 |
| Lateral Occipital Cortex | L | 193 | .015 | 4.35 | -22 | -66 | 32 |
| Threat First > Protection First | | | | | | | |
| Lateral Occipital Cortex | R | 204 | .012 | 4.56 | 48 | -74 | 4 |
| Protection Second > Threat Second | | | | | | | |
| Occipital Pole | L | 4111 | 3.30 x 10^-22^ | 7.01 | -16 | -90 | -16 |
| Postcentral Gyrus | L | 748 | 7.75 x 10^-07^ | 4.94 | -40 | -42 | 66 |

Note. *N* = 33. Cluster-defining threshold *Z* > 3.1, FWE-corrected *p* < .05. R = right hemisphere, L = left hemisphere. Cluster peak regions are based on the Harvard-Oxford Structural Atlas. Local maxima not listed.

## Run-Wise Habituation Analysis

To rule out the habituation effect across the four runs of the Safety Estimation Task, we extracted run-specific estimates for the Protection First > Threat First contrast from our three a priori ROIs (ACC, anterior vmPFC, Hippocampus; see Fig. S1) and conducted repeated-measures ANOVAs. There was no main effect of run for the hippocampus (F = 1.32, p = .274), anterior vmPFC (F = 0.28, p = .809), or ACC (F = 0.97, p = .409). These results indicate no evidence of habituation across the task.

**Figure S1**

Run-wise ROI Activation for **Protection First > Threat** First (ACC, anterior vmPFC, Hippocampus)
